# Experimental evolution reveals microbial traits for association with the host gut

**DOI:** 10.1371/journal.pbio.3000129

**Published:** 2019-02-04

**Authors:** Nicole M. Vega

**Affiliations:** Department of Biology, Emory University, Atlanta, Georgia, United States of America

## Abstract

Understanding how microbes adapt to their host is an enduring problem in microbiome ecology, and understanding the microbial traits that allow colonization of the host and increase adaptation to the host environment is of particular interest. In this study, Robinson and colleagues use experimental evolution to demonstrate adaptation of a commensal bacterium to its zebrafish host and describe the changes in phenotype that emerge during this evolutionary process. These results provide insight into the evolutionary problem of host adaptation and demonstrate the utility of simple models for understanding host–microbiome dynamics.

Increasingly, host-associated microbial communities are being viewed through an ecological lens [1–3]. On one level, the search for “core” microbiomes seeks to define the composition of “normal” host-associated communities and to identify the functions they perform [4]. Moving deeper, these primarily observational studies are increasingly supplemented by experiments designed to determine the interactions among microbes, and between microbes and host, that shape and stabilize a microbiome.

A natural question is—what makes these communities particularly suited to their hosts? In short, how do the members of a microbiome become members of that microbiome? The gut microbiome of an organism is characteristic of that organism, featuring an identifiable profile of clades [5,6] and an internally normal spectrum of diversity. We tend to assume, not unreasonably, that this is the result of coevolution between microbes and their host. Experimental evolution studies allow researchers to observe the evolution of traits in real time. It is reasonable to ask, therefore, what ecological and evolutionary forces are needed to adapt a microbe to its host environment.

The environment presented by a given host is very important for selecting the strains that will be able to colonize and thrive as part of its microbiome [7]. This suggests, first, that environmental filtering—in which the environment selects for or against certain species—is an important driver of community assembly in the host [8,9] and, second, that selective pressure after filtering could increase adaptation of microbial strains to the host environment [10,11]. In fact, adaptation of strains to the environment presented by an individual [12] may be important for retention of specific lineages over time.

There is no doubt that adaptation to the host can greatly facilitate colonization by a given microbe [13], and indeed, some very well characterized examples exist. Adaptation of microbes to the particular nutrient sources and physical habitats available within a host can increase a microbe’s ability to colonize and compete with other bacteria [14,15], as can weaponry for direct competition between microbes [16], suggesting that victory over competitors is important for success in these communities. Furthermore, modulation of host immunity can improve colonization by commensal gut bacteria [17]. However, for the greater bulk of host-associated microbes, and particularly for those that transition from host to host by traveling through the external environment, the evolution of host adaptation is not well understood.

Experimental microbial evolution has increasingly been used as a tool to understand how microbiome-associated bacteria come to occupy their particular host [18]. Observational studies comparing the genomes of host-associated microbes against their environmentally associated relatives can be very useful for finding genes and mutations associated with particular instances of host adaptation, for example, in describing the mechanisms of commensalism for an important human symbiont [11]. However, these studies have the disadvantage that disentangling host-adaptive mutations from other mutations accumulated over time can be a challenge. Furthermore, advantages in fitness are a function of organismal traits; a given phenotype may be arrived at via multiple mutations, and it can be challenging to map observed mutations to the advantageous traits they control. By contrast, experimental evolution allows researchers to trace adaptation precisely by preserving microbial lineages over the course of an experiment [19] and to observe what phenotypes emerge in multiple independent lineages to better understand what traits are being selected for during evolution in the host.

In this study, Robinson and colleagues [20] use experimental evolution to show how a bacterial isolate (*Aeromonas veronii*) becomes increasingly better adapted to colonization of its animal host (the zebrafish) by repeatedly moving it from host to host through the external water environment. Though originally isolated from the zebrafish gut, this *Aeromonas* was a relatively poor colonizer as compared with many other gut isolates [21,22], suggesting that there was room for evolution to shape this organism into a more prolific colonizer. To this end, an engineered strain of *Aeromonas* with a high mutation rate (Aer01) was introduced to batches of germ-free larval zebrafish, in which it was taken up and colonized the intestine; the intestines of colonized fish were then dissected, and the gut contents were used to inoculate the environment of the next generation of hosts. This experimental design allowed Aer01 the chance to evolve by repeatedly cycling between the environmental reservoir and the host gut.

Over time, Aer01 was expected to adapt to increase its association with the host. The question was—how? A number of traits were potentially under selection (Fig 1). Aer01 could increase its presence in the gut, for example, by increasing its growth rate or total abundance in the intestine, becoming more resistant to expulsion, or increasing its rate of transit into the gut from the environment. Alternately, increasing persistence and/or population in the external environment could increase entry into hosts without involving host-specific adaptation at all.

**Fig 1:**
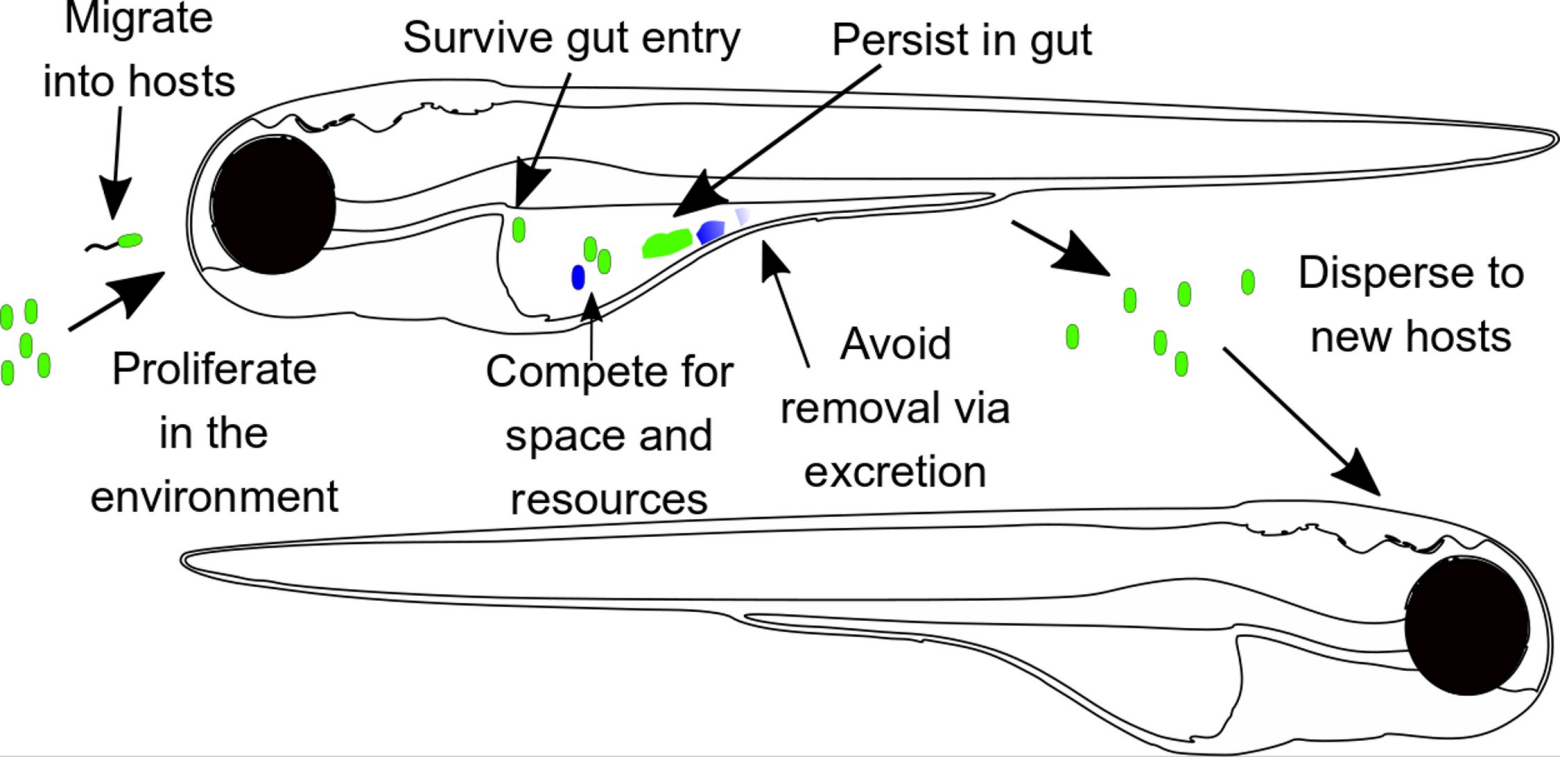
Multiple selective pressures can shape host adaptation of a microbe. An evolved commensal (green) can outcompete its unevolved ancestor (blue) by evolving a selective advantage in one or more of a number of traits relevant to entry into the host, survival and retention in the host environment, and/or transit between individual hosts through an environmental reservoir. Larval zebrafish image derived from original image by Lizzy Griffiths (http://zebrafishart.blogspot.com).

Robinson and colleagues found that Aer01 consistently evolved a higher rate of transit into the gut from the environment. Mechanistically, this change occurred by increasing bacterial motility, which resulted in increased competitive ability in the gut as compared with the ancestral strain. The advantage only manifested when Aer01 was allowed to enter the host naturally from the environment, rather than by gut or mouth gavage, suggesting that selection acted to specifically improve Aer01 entry into the gut from the environment.

Following the initial innovation of improved colonization, these strains continued to increase their competitive ability in ways that were specific to the host. These further-evolved isolates were able to outcompete their ancestors even more effectively—but only in the host lineage used for evolution. When an immune-compromised host was used in place of the wild-type host, the advantage of this additional evolution disappeared.

These results suggest a conserved trajectory for evolution in this system. First, Aer01 evolved an improved ability to enter the host from the environment. The zebrafish gut is dynamic, characterized by periodic sudden population collapses [21], which open up large chunks of available space to the next successful colonizer, making rapid colonization advantageous. If other host-associated microbiomes share this boom-and-bust dynamic, this strategy may prove to be common when an environmentally acquired microbe is undergoing adaptation to its host. Although Aer01 apparently evolved specifically to increase transit from the external environment into the gut, other phenotypes could also increase colonization rate—decreased bottlenecking during transit from the mouth into the gut, for example, or increased residence in the host intestine. (Microbes in the zebrafish gut do not adhere to the intestinal epithelium [23,24], and so evolution of residence time would have to occur through a different mechanism.) It remains to be seen why Aer01 evolved the specific adaptation that it did and whether other strains faced with the same problem will come up with alternate solutions.

Increased host-to-host transmission is part of the selective advantage enjoyed by the evolved strains. Interhost dispersal is known to be a major force shaping the gut community in zebrafish [25], and migration may be a major contributor to population dynamics in host-associated microbial systems, particularly during initial colonization [26–28]. This suggests that migration may be a major hurdle to be cleared in the course of host adaptation. It will be interesting to see whether experimental evolution in other host–microbe combinations produces similar results.

Another component of this selective advantage arose during adaptation to the specific host environment—in particular, to the wild-type immune system. This suggests that host immunity posed a considerable problem to this colonist—or a great opportunity. At present, the mechanism of interaction between Aer01 and the zebrafish immune system is not clear, but uncovering the specifics of this interaction will provide considerable insight into the particular problem being solved by a microbe as it becomes host adapted—whether, for example, Aer01 has managed to evolve internal defenses against the stress of host immune attack, to regulate the expression of intestinal immunity by the host, or something else entirely.

Although this work focused on evolution of a single microbial strain for reasons of tractability, the polymicrobial environment is more typical for host–microbe associations. Interactions between microbes can alter the trajectory of evolution in the host environment, even shaping pathogens into commensals [29] (and presumably vice versa, if the right circumstances can be found). However, the natural gut microbiome of an animal may contain tens or thousands of clades, and determining which interactions are important in these large communities in the spatially structured environment of a host gut is far from trivial. There is a good deal of interest in untangling the interactions that determine the ecology of these communities [30–32], which may provide a foundation for understanding their evolution, but a full understanding of the ecological and evolutionary dynamics within the massive, shifting ecosystem of the mammalian gut may yet be some time off.

This work was designed to provoke evolution of bacteria to increase association with the host but did not go so far as to permit coevolution of the host with this microbe; this represents an intriguing possible future direction. The use of small model hosts with shorter generation times, such as nematodes and *Drosophila*, will allow experimental evolution to take into account coevolution between microbe and host, and coevolution can readily alter the evolutionary trajectories in these systems [33]. It will be very interesting to see the outcomes of coevolution of microbe and host during “capture” of an environmentally acquired commensal, to clarify the mechanisms at work on both sides of the process.

In this manuscript, Robinson and colleagues have provided a fascinating glimpse into the evolution of a host-associated microbe, demonstrating the phenotypes that provide an advantage to the bacteria as they adapt to associate with their host. They demonstrate an evolutionary trajectory that is apparently contingent on phenotype, in which the initial innovation of increased motility allows increased association with the host, which in turn provides the basis and opportunity for host-specific evolution. Using a tractable experimental system, this work provides an accessible template for understanding the evolution of a host-associated microbe, as well as insights into the importance of the environmental reservoir during evolution of a horizontally transferred commensal. This work will serve as a foundation for future work using experimental evolution to understand how microbes navigate the transition from environment to host.
